# 

**Published:** 1865-12

**Authors:** 


### CONTENTS.

### ORIGINAL ESSAYS AND COMMUNICATIONS.

Dental Materia Medica ... 487
Address, read before the Merrimack Valley Dental Association, by A. Lawrence ... 492
Are you a Reading Man ... 496
Address, before the Cincinnati Dental College Society, by Wm. Taft ... 502
Steam Gauge ... 505
Address, read before the Brooklyn Dental Association, by G. A. Mills ... 507
Extracting Teeth ... 511
Remarks on the Institutes of Dental Science, by C. W. Spalding, D. D. S. ... 513
A Short Review ... 517

### PROCEEDINGS OF SOCIETIES.

Connecticut Valley Dental Association ... 516

### SELECTIONS.

Soft Sarcomatus Tumor of the Antrum of Highmore ... 518
Source of the Phosphates in Bone ... 520
Abuse of Alcohol, ... 521
Influence of Mental Impressions as a cause of Bodily Deformity ... 523
Means of averting Death from Chloroform ... 525
Danger of Subcutaneous injections ... 526
New Method of Disinfecting and Deodorizing ... 527
Destruction of Tumors by Electricity ... 527
Salivary Calculi, ... 528
Deglution ... 528
Surgery v. Medicine ... 529

### EDITORIAL.

Close of the Volume ... 529
Back Numbers ... 530
The Twentieth Volume ... 530
A Case ... 531

